# Editorial: Novel trends in cultured meat research

**DOI:** 10.3389/fnut.2024.1452643

**Published:** 2024-07-16

**Authors:** Sishir K. Kamalapuram, Marie-Pierre Ellies-Oury, Sghaier Chriki, Jean-François Hocquette, Andrew C. A. Wan, Ivana Gadjanski

**Affiliations:** ^1^Sanjeevani Bioservices Pvt, Ltd, Hyderabad, India; ^2^Bordeaux Sciences Agro, INRAE, Gradignan, France; ^3^Institut national de recherche pour l'agriculture, l'alimentation et l'environnement (INRAE), Université Clermont Auvergne, VetAgro Sup, UMR1213, Recherches sur les Herbivores, Saint Genès Champanelle, France; ^4^Institut supérieur d'agriculture Rhône-Alpes (ISARA), Institut national de recherche pour l'agriculture, l'alimentation et l'environnement (INRAE), Lyon, France; ^5^Singapore Institute of Food and Biotechnology Innovation (SIFBI), Agency for Science, Technology and Research (A^*^STAR), Singapore, Singapore; ^6^BioSense Institute - Research and Development Institute for Information Technologies in Biosystems, University of Novi Sad, Novi Sad, Serbia

**Keywords:** cell-based food, bioreactors, scaffolds, skeletal muscle satellite cells, cell expansion, consumer behavior

According to a rather large number of scientists, investors and stakeholders, cultured meat (CM) industry is on the cusp of a revolution, growing at rapid pace and is expected to be a significant player in the global meat market in the near future (1). As the CM industry rapidly evolves, it is essential to critically examine the scientific underpinnings and obstacles encountered in the use of bioreactors, scaffolds including nanofibers, microcarriers, etc. for CM cell expansion (2), the urgent need for single-cell analysis of CM cell types (3) and issues related to genetic drift and stem cell adherence during CM cell growth (3, 4). Further, understanding consumer attitudes and preferences toward future CM products is another important aspect that will certainly aid the CM sector move in the right direction (5).

Our Research Topic on “*Novel trends in cultured meat research*” had published seven articles addressing the most critical and recent developments in global CM sector in following order as detailed below.

Jacobs et al. conducted an online survey among 3,558 potential German adult consumers, in order to identify the trends in terms of willingness to try, regularly eat, or pay for CM products. The vast majority (70%) stated that they would be willing to try it, with the most important drivers being ethics, curiosity and eco-friendliness. Around 57% of the participants said they would be willing to eat artificial meat regularly. Most of the respondents (40%) were willing to pay the same price for artificial as for conventional meat. CM was considered as promising and acceptable (63%), more ethical (67%) and more environmentally friendly (58%) by survey respondents in comparison to conventional meat whereas respondents from other countries (using the same survey) as well as the scientific community are not so optimistic [Jacobs et al.; (6)].

Hauser et al. in their review article, detailed critical cellular mechanisms underlying proliferation rates and also addressed crucial intrinsic limitations such as effective functioning of cell cycle regulators that coordinate cell signaling pathways and conditional rate limitations including temperature, nutrient optimization, pH and oxygen levels for CM cell expansion. The article presented prospective CM cell expansion strategies involving genetic modification of selective genes that can immortalize cells, increase cell proliferation, and also facilitate adaptation of CM cells to various media formulations and cell culture techniques. This can promote enhanced overall scalability and significant cost-efficiency, during phases of CM cell proliferation. Further, the article detailed various tissue engineering strategies including 3D cell culture, basic cellular signaling mechanisms such as Cyclin-dependent kinase (CDK), Phosphatase and tensin homolog (PTEN), P53 pathways and their pathological contexts, that can enable identification of effective and innovative solutions for optimization of CM cell expansion (Hauser et al.).

Santos et al. developed a transformative scaffold by utilizing random cellulose acetate nanofibers (CAN) for muscle tissue engineering (MTE) applications. C2C12 and H9c2 chicken myoblast cells were grown on random and aligned CAN. Further, comparative analysis of both CANs revealed that random CAN facilitated muscle differentiation through YAP/TAZ-related mechanotransduction pathway, irrespective of differentiation media. These C2C12 and H9c2 myoblast-loaded CAN sheets were utilized to create a three-dimensional meat product stacked as a four layered tissue of 2 cm length and 300–400 μm thickness, arranged in the form of a mesh of uniaxial aligned cells. This biomimetic strategy is potentially cost-effective for cultivation and differentiation of muscle cells (Santos et al.).

An insightful review article on CM cell expansion by Kulus et al. identified cell sources including adult stem cells, adult somatic cells, embryonic stem cells, and fibro/adipogenic progenitor cells for CM. This article presented in-depth strategies for overcoming current obstacles related to—(a) bioreactors: achieving optimized cellular growth rates in batch, fed batch and continuous bioreactor setups, (b) scaffolds: utilization of non-toxic, biodegradable scaffolds that promote enhanced CM cell differentiation, favorable cell arrangement providing optimal shape to the CM end product, (c) microcarriers: for achieving high density cell adherence to microcarriers and high surface area to volume ratio in bioreactors for CM cell expansion, (d) potential societal barriers: importance of cultural preferences and consumer behavior for promoting CM as a viable food source are discussed (Kulus et al.).

Messmer et al. utilized single-cell RNA sequencing strategy to identify cellular heterogeneity patterns in bovine skeletal muscle satellite cell (MuSCs) cultures for development of cell purification and proliferation protocols and further profiled distinctive cell types. Overgrowth of undesirable cells in a heterogeneous cell population has been identified as a potential barrier to efficient CM production. Transcriptomics approach was employed to uncover favorable growth conditions of MuSCs in serum-free medium. RNA velocities computed *in silico* in combination with time-resolved flow cytometric analysis were employed to understand dynamic subpopulations and various transition stages including active, quiescent, and committed state of MuSCs. Moreover, methodology for modulating of transient stages of MuSCs, during long-term proliferative cultures was demonstrated. Overall, this novel research can be an important reference for advancing knowledge in bovine skeletal muscle biology for CM development (Messmer et al.).

Tzimorotas et al. studied the long-term, large scale cell growth strategies for bovine skeletal muscle satellite cells (MuSCs) by employing low-volume spinner flasks and benchtop stirred-tank bioreactor (STR). Significant growth of primary MuSCs was noted in a bench-top STR run with a lesser initial cell seeding density, low glucose and also FBS reduction for a time period of 38 days, which is significantly longer than in any previous studies on bovine MuSCs. Further, enhanced expression of the paired box protein 7 (PAX7) satellite cell growth marker and reduced levels of differentiation-inducing myogenic factor like MYOG was observed, even without addition of p38-MAPK cell growth inhibitors, resulting in progressive growth of MuSCs. Overall, bovine MuSCs grown in lab-bench bioreactors exhibited significant proliferation, migration and differentiation potential over an extended period of time (Tzimorotas et al.).

In their review article, Jaime-Rodríguez et al. discussed the major challenges of genetic drift and adherence of mesenchymal stem cells during large scale CM production. Genetic drift can be caused when cells are exposed to selective pressure, leading to oncogenic gene activation and loss of their stem cell characteristics. Hence, the dire need for genetic and functional analysis of cells, in order to determine the maximum number of cell passages without detection of mutations and functional loss of stem cell potential was detailed. The other challenge discussed was mesenchymal stem cell adherence in bioreactors, which can be hindrance for their large scale proliferation, due to the limited volume to surface ratio in high volume cell culture containers. Prospective solutions such as edible microcarriers, multi-tray systems, roller bottles were discussed, to address this limitation and also minimize the downstream operations in large scale CM production (Jaime-Rodríguez et al.).

In conclusion, we hope that our Research Topic will provide information regarding the latest technical developments in the global CM sector, obstacles encountered during CM production and their potential solutions. Despite limited contributions in this area, we also hope that this Research Topic will contribute to highlighting the importance of understanding consumer behavior for the potential implementation of the CM industry. This is indeed a key issue to be further studied.

## Author contributions

SKK: Writing – original draft, Writing – review & editing. M-PE-O: Writing – review & editing. SC: Writing – review & editing. J-FH: Writing – review & editing. AW: Writing – review & editing. IG: Writing – review & editing.
